# Account of a Peculiar Kind of Leech, Swallowed and Stopped in Different Parts of the Throat

**Published:** 1803-03-01

**Authors:** 


### *Account of a peculiar Kind of* LEECH, *swallowed and stopped in different Parts of the Throat*; *by* Cit. LARREY.

THE worms, which are the subject of this observation, live in pools of muddy water, in the middle of those deserts which separate Egypt from Syria, and of those on the confines of Lyba; they have the form of a horse hair, and are some lines only in length; but filled with blood they become the size of an ordinary leech. When the French army entered this country, the soldiers, pressed by thirst, threw themselves on their mouth and nose, and drank greedily of this water; many of them felt immediately, stings or prickling pains in the posterior fauces, followed by frequent cough, glairy spots lightly tinged with blood, and a disposition to vomit; a difficulty of swallowing, laborious respiration, and sharp pains in the chest: The patient lost his appetite and rest, became then uneasy and agitated, and if the complaint was not relieved he fell a victim. The first person attacked thus, besides those symptoms, had lost much blood. On coming into the hospital, C. LARREY, on pressing down the tongue with a spoon, perceived the leech, which was of the size of the small finger. He introduced a small forceps to lay hold of it; but on the first touch it contracted, and placed itself behind the velum pendulum palati.

As soon as it had resumed its former position he seized it with a polypous forceps; the consequent hæmorrhage soon ceased, and the soldier was perfectly well in a few days. About twenty soldiers were attacked in the same way on the march of the army from Syria to Belbeis. Gargles of vinegar and salt water were sufficient to detach these animals, who placed themselves constantly on the posterior fauces: Fumigations of tobacco, and the polypous forceps were necessary in some cases. The chief of brigade, Lateur Mauberg, commander of the twenty-second regiment of Chasseurs, swallowed two in the deserts of St. Makaire, a day's journey from the pyramids. They reduced him to the last state of emaciation and weakness; and even after detaching these animals, the convalescence was long and difficult. Cit. Larrey gives many other cases of the same kind, in the Memoir from which this Extract is taken; he recommends travellers through these deserts, who should be obliged to drink this water, and in which the presence of these animals is to be apprehended, to strain it through a thick and close cloth, and to add some drops of any acid.

